# Epidemiological study of *Trichosporon asahii* infections over the past 23 years

**DOI:** 10.1017/S0950268820001624

**Published:** 2020-07-24

**Authors:** Haitao Li, Meihong Guo, Congmin Wang, Yibo Li, Anne Marie Fernandez, Thomas N. Ferraro, Rongya Yang, Yong Chen

**Affiliations:** 1 Department of Dermatology, The Seventh Medical Center of PLA General Hospital, Dongcheng, 100700, Beijing, China; 2 Department of Biological Sciences, Center for Systems Biology, the University of Texas at Dallas, Richardson, TX 75080, USA; 3 Department of Molecular and Cellular Biosciences, Rowan University, Glassboro, NJ 08028, USA; 4 Department of Biomedical Sciences, Cooper Medical School of Rowan University, Camden, NJ 08103, USA

**Keywords:** Clinical characterisation, fungi infection, therapeutic prognosis, *Trichosporon asahii*

## Abstract

Trichosporon is a yeast-like basidiomycete, a conditional pathogenic fungus that is rare in the clinic but often causes fatal infections in immunocompromised individuals. *Trichosporon asahii* is the most common pathogenic fungus in this genus and the occurrence of infections has dramatically increased in recent years. Here, we report a systematic literature review detailing 140 cases of *T. asahii* infection reported during the past 23 years. Statistical analysis shows that *T. asahii* infections were most frequently reported within immunodeficient or immunocompromised patients commonly with blood diseases. Antibiotic use, invasive medical equipment and chemotherapy were the leading risk factors for acquiring infection. *In vitro* susceptibility, clinical information and prognosis analysis showed that voriconazole is the primary drug of choice in the treatment of *T. asahii* infection. Combination treatment with voriconazole and amphotericin B did not show superiority over either drug alone. Finally, we found that the types of infections prevalent in China are significantly different from those in other countries. These results provide detailed information and relevant clinical treatment strategies for the diagnosis and treatment of *T. asahii* infection.

## Introduction

Over the past 30 years, the incidence of fungal infection has increased dramatically in patients with cancer, deep burns, organ transplants and those under treatment with hormones and immunosuppressive agents, becoming one of the main causes of death in these individuals. In addition to common fungal infections such as candidiasis and aspergillosis, infections caused by non-candida yeast such as trichosporonosis are increasing in immunosuppressed, immunocompromised and immunodeficient individuals with severe conditions and high mortality rates [1–4]. Trichosporon is a yeast-like basidiomycete. It is ubiquitous in nature meaning it is present in soil, water, plants, mammals, birds, etc. and is also present among the human body's natural flora; however, it can become pathogenic to the humans in certain cases [5–8]. Hermann Beigel first proposed the concept of Trichosporon in 1865 and observed that it can cause hair infection. The classification and naming of Trichosporon has then changed multiple times over the years. Initially, all *Trichosporon* spp. were classified as *Trichosporon beigelii*, and it was thought that the strain mainly causes infection in superficial hair, and rarely causes deep-seated infection [6]. However, subsequent research showed that *T. beigelii* has great morphologic variation, as well as physiological and biochemical variance [9]. In 1992, Gueho *et al*. revised the taxonomy of 20 species of Trichosporon, including six pathogens: *Trichosporon asahii*, *T. asteroides*, *T. cutaneum*, *T. inkin*, *T. mucoides* and *T. ovoides* [6, 10–12]. In 1994 and 1995, Sugita *et al*. reviewed the genus *Trichosporon* and proposed a new classification that included 17 different species of *Trichosporon* and five varieties [13]. By 2002, the number of Trichosporon species had increased to 25, of which eight were associated with human infections, including two new species, *T. domesticum* and *T. montevideense* [14]. Presently, 50 Trichosporon species are recognised, of which 16 are known to be pathogenic [15–19].

Current research indicates that risk factors for Trichosporon infection include neutropaenia (an abnormally low amount of neutrophils), organ transplantation, diabetes, end-stage renal disease, HIV infection and use of immunosuppressive agents and invasive medical equipment [5, 6, 20–27]. Trichosporonosis is associated with a variety of infection types, including superficial skin and hair infections, summer-type hypersensitivity pneumonitis (SHP), chronic pneumonia, meningitis, endocarditis, disseminated infections and fungaemia [28–41]. Trichosporon infections are also often misdiagnosed as other types of fungal infections [42], and have proven difficult to treat. Before 2000, physicians recommended amphotericin B for treatment of Trichosporon infections; however, usage of this drug has been reported to be ineffective [43]. The combination of amphotericin B and flucytosine was also suggested for treatment [37, 44, 45], but no studies are published that compare effectiveness between combined and individual antifungal drug treatment. When azole pharmaceuticals appeared on the market (especially voriconazole), most *in vitro* susceptibility testing showed that they were superior to amphotericin B for treating trichosporosis [46–48]; however, the *in vitro* effects of antifungal drugs are not fully representative of their therapeutic effectiveness in humans. The guidelines developed by ESCMID/ECMM in 2014 recommend voriconazole for the treatment of trichosporosis, although it is noted that the development of this guide relies mainly on *in vitro* susceptibility testing results, animal model test results and a few case reports [49].

Almost all cases of Trichosporon infection reported before 1994 were named *T. beigelii*. In contrast, isolates of *Trichosporon* spp. reported in the past 20 years are of various types, and numerous species of Trichosporon are known to exhibit different pathogenic characteristics. For example, *T. asteroids* and *T. cutaneum* are the main pathogenic species known to cause superficial skin infections [6]. On the other hand, *T. asahii* is the most prominent clinical pathogen in the genus. *T. asahii* causes more than half of the infections of the genus [50], and the mortality caused by deep invasive infections is greater than 70%. In 1992, Gueho *et al*. [6] proposed another classification of *Trichosporon* spp., and confirmed that *T. asahii* was the main pathogen causing deep trichosporosis.

The incidence of *T. asahii* infection has increased significantly in the past 20 years [1, 3]. It is commonly found in immunodeficient and immunocompromised patients, and individuals with blood diseases [23, 51, 52]. A few studies also reported *T. asahii* infection in healthy individuals [21, 22]. In addition, different species of Trichosporon possess different drug sensitivities. For example, amphotericin B has a higher minimum inhibitory concentration (MIC) against *T. asahii*, *T. faecale* and *T. coremiiforme*, while *T. inkin*, *T. ovoides*, *T. japonicum*, *T. domesticum*, *T. montevideense* and *T. cutaneum* have lower MICs [46, 53–56]. Therefore, it is important to accurately analyse the epidemiological characteristics of different species of Trichosporon so the susceptibility and risk factors of infection can be obtained. This information can then enhance patient diagnosis, treatment, medication and outcome prediction.

Accurate epidemiological analysis of Trichosporon infections, especially *T. asahii*, can highlight susceptibility to infection and improve patient diagnosis, treatment and prognosis. Although there is some literature reviewing Trichosporon disease [57–59], a comprehensive analysis of epidemiological characteristics, clinical treatment and prognosis of *T. asahii* infection has not been published. Here we systematically analysed the epidemiological characteristics, risk factors, clinical manifestations, diagnosis, treatment, medication and prognosis of *T. asahii* infections reported in the English literature from 1996 to 2019 and in Chinese literature from 2009 to 2019. We then compared the infections in China with those in other countries to determine significant differences in divergent clinical categories.

## Materials and methods

### Literature collection

In order to do a comprehensive analysis of clinical studies regarding *T. asahii* infections, we performed a systematic literature review to identify original cases in both English and Chinese literature. First, we reviewed all references from the PubMed database by using the keywords *T. asahii*, *trichosporon asahii* and *trichosporonosis*. The search was then limited to studies including human subjects, clinical trials and case reports. A search was also conducted on the Chinese medical database WANFANG (http://www.wanfangdata.com.cn/index.html). To standardise analysis, the following patient information was examined for each case: time of onset, study area, demographic data, possible pathogenesis, type of underlying disease, source of specimen, identification method of strain, type and location of infection, treatment and outcome. Published studies with missing case information from any of these categories were excluded. All literature reviewed was published between 1996 and 2019.

### Statistical analysis of epidemiological data

For each category, the distribution of data was analysed by using a descriptive statistical analysis. To understand the different causal factors and therapy results between 51 Chinese cases and 89 cases from other countries, we compared each cohort with respect to the patient information. A Fisher's exact test was used to compare the effectiveness of the three antifungal drug groups (amphotericin B; triazoles; amphotericin B plus triazoles), where *P* < 0.05 was considered statistically significant.

## Results

### Patient population

**Fig. 1.**
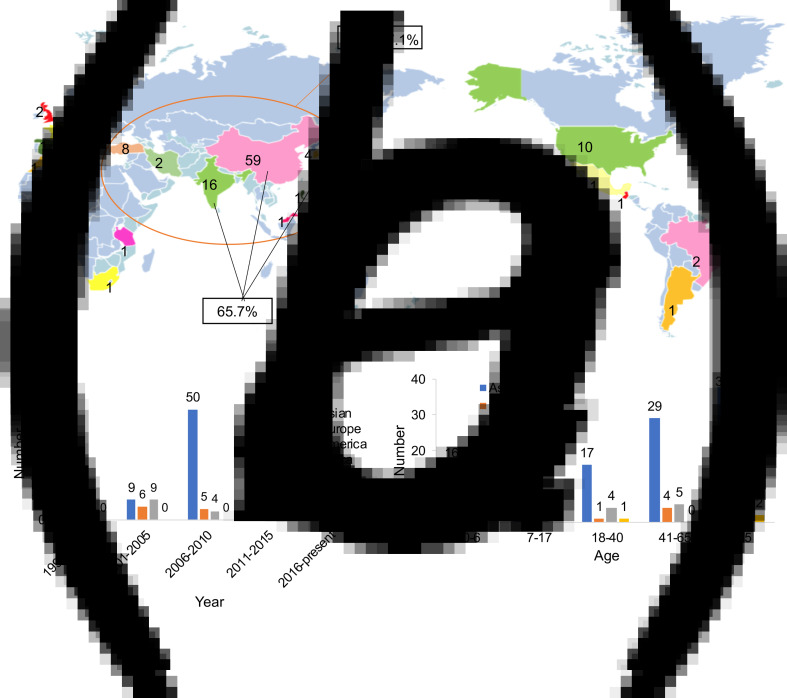
Basic statistical features of 140 cases of *T. asahii* infection. (a) Regional distribution. (b) Temporal distribution of 140 cases (year). (c) Age distribution.

Systematic literature review yielded a total of 140 cases of *T. asahii* infection, including 51 cases in Chinese literature and 89 in English literature reported from 1996 to 2019. These cases were distributed throughout Asia, Europe, North and South America and Africa, in which Asia had the largest proportion (108/140, 77.1%). Cases reported from China, Japan and India represented 65.7% of the total. Among other countries and regions, 10 cases were reported from the United States (Fig. 1a). The number of cases in Asia dramatically increased from 2006 to 2015, but only 16 cases were observed in other years (Fig. 1b).

Of the 140 patients with *T. asahii* infection, 83 were male (83/140, 59.3%), 47 were female (47/140, 33.6%) and the gender of remaining 10 patients was not reported (eight were newborn premature infants), achieving a ~1.77-fold ratio of males to females. The age of onset ranged from 0 to 92 years old, where the group of individuals over 65 years old represented the largest proportion (48/140, 34.3%). For cases from Asia, the older individual groups have higher proportions of *T. asahii* infection, which is also consistent with observations from Europe and the Americas (Fig. 1c).

### Risk factors for *T. asahii* infection

**Fig. 2.**
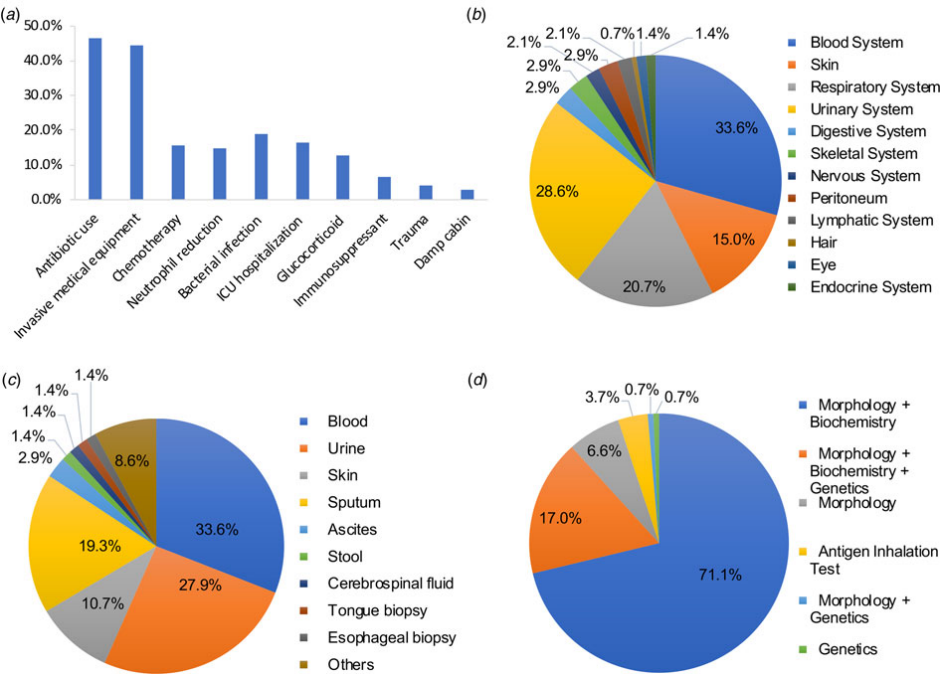
Proportions of 140 cases on risk factors (a), infected tissues (b), test samples (c) and testing methods (d).

Previous studies found divergent risk factors related to trichosporosis including antibiotic usage, invasive medical equipment, intensive care unit (ICU) hospitalisation and others [60, 61]. Among the 140 patients with *T. asahii* infection, we also found that antibiotic and invasive medical equipment users were the two major groups with proportions of 46.4% (65/140) and 44.3% (62/140), respectively (Fig. 2a). Overall, the use of broad-spectrum antibiotics, the use of invasive medical devices, neutropaenia and ICU hospitalisation remain the four greatest risk factors for *T. asahii* infection. Our results proved to be consistent with previous studies. For example, Kontoyannis *et al*. [61] analysed 17 patients with trichosporosis and a history of oncology and found that 12 patients used central venous catheters and 11 patients had neutropaenia. Ruan *et al*. [60] analysed 19 patients with disseminated trichosporosis infections in Taiwan between 2000 and 2008, and found that 18 patients used central venous catheters and 17 patients used broad spectrum antibiotics.

We further checked if prior antifungal treatment/prophylaxis is a risk factor for *T. asahii* infection. Based on the previous definitions of breakthrough invasive fungal infection (IFI) [62], a total of 22 cases can be classified as breakthrough IFI during prophylaxis or empiric treatment (Table 1). Among them, amphotericin B (27.3%), echinocandin (22.7%) and fluconazole (13.6%) are three top used antifungal drugs with regard to risk for breakthrough IFI. Furthermore, IFI was also observed after prophylactic use of antifungal drugs in combination, e.g. fluconazole plus amphotericin B (9.1%). After targeted antifungal treatment of *T. asahii* infection, nine patients were cured but 13 died.

**Table 1.** Statistical summary of 22 breakthrough IFI with prior antifungal treatment/prophylaxis

Prophylactic antifungal drugs	No.（%）	Citations (PMID)
Echinocandin	5 (22.7)	24383917, 23419599, 25328390, 17882360
Amphotericin B	6 (27.3）	26351595, 18362402, 12087540, 16964817, 16217998
Fluconazole	3 (13.6)	22370746, 20436525, 11337182
Itraconazole	2 (9.1)	17322000, 16838224
Posaconazole	1 (4.6)	17690928
Fluconazole + micafungin	1 (4.6)	15231763
Fluconazole + amphotericin B	2 (9.1）	25383317, 11477533
Fluconazole + itraconazole + amphotericin B	1 (4.6）	23970618
Unknown	1 (4.6)	18356615

### Underlying disease types

Haematologic diseases (39/140, 27.9%), diabetes (19/140, 13.6%) and pulmonary diseases (17/140, 12.1%) were the three most common pre-existing illnesses in patients within the 140 *T. asahii* infection cases. Among the patients with blood diseases, 28 patients had leukaemia, accounting for 71.8%. There were also patients with other maladies and conditions such as AIDS, organ transplants and various cancers. Interestingly, five patients were apparently healthy prior to infection without any pre-existing illnesses (Table 2). The distribution of underlying diseases among *T. asahii* infections differs from the distribution observed among general Trichosporon infections. In a study of underlying diseases in patients with Trichosporon infection, Girmenia *et al*. [57] reported that the three most common pre-existing clinical conditions were haematological diseases, peritoneal dialysis and malignant tumours. Our study also showed that in patients with *T. asahii* infection, blood diseases (mainly blood cancer and neutropaenia) are most common, diabetes is second and lung disease follows as third.

**Table 2.** The underlying disease types of 140 patients^a^ with *T. asahii* infection

Types of underlying disease	No.（%）	Types of underlying disease	No.（%）
Blood disease	39 (27.9)	Malignant tumour	4 (2.9)
Diabetes	19 (13.6）	Fracture	4 (2.9)
Lung disease	17 (12.1)	AIDS	3 (2.1)
Other	14 (10)	Organ transplantation	3 (2.1)
Premature infants	10 (7.1)	Chronic gastritis	3 (2.1）
After surgery	7 (5)	Hypothyroidism	2 (1.4)
Chronic renal insufficiency	6 (4.3)	Rheumatoid arthritis	2 (1.4)
Summer allergic pneumonia	4 (2.9)	Diabetic nephropathy	1 (0.7)

a Some patients have more than one underlying disease.

### Clinical manifestations, type of infection and site of infection

The clinical manifestations of *T. asahii* infected patients were non-specific, but varied with different infection sites and types of infections. The major types of infections were urinary tract infection (35/140, 25%), fungaemia (33/140, 23.6%) and disseminated infection (20/140, 15.7%). A few other infections reported were SHP, peritonitis, infections found superficially on the skin and in hair, along with various other infection types (Table 3). Among the 140 patients with *T. asahii* infections, infection of the blood was the most common (47/140, 33.6%), followed by the urinary system (40/140, 28.6%), respiratory system (29/140, 20.7%) and integumentary system (21/140, 15.0%) (Fig. 2b).

**Table 3.** Clinical illnesses in patients with *T. asahii* infection

Type of infection	No.（%）	Type of infection	No.（%）
Urinary infection	35 (25)	Glossitis	1 (0.7)
Fungaemia	33 (23.6)	Conjunctivitis	1 (0.7)
Disseminated infection	20 (15.7)	Discitis	1 (0.7)
Lung infection	15 (10.7)	Infective endocarditis	1 (0.7)
Skin infections	8 (5.7)	Black hairy knot disease	1 (0.7)
Chronic pneumonia	7 (5)	Lymphadenitis	1 (0.7)
Other	4 (2.9)	Endophthalmitis	1 (0.7)
Peritonitis	3 (2.1)	Brain abscess	1 (0.7)
Meningoencephalitis	2 (1.4)	Synovitis	1 (0.7)
Esophagitis	2 (1.4)	Summer allergic pneumonia	1 (0.7)

### Strain identification methods

*T. asahii* was isolated from a variety of clinical specimens. Of the 140 patients studied, 47 were diagnosed using a specimen obtained from blood (33.6%), 39 were diagnosed from a urine specimen (27.9%) and 27 were diagnosed from a sputum specimen (19.3%). A complete description of samples used for testing can be found in Figure 2c. Of the 140 *T. asahii* cases, 135 cases (96.4%) described the method for identifying *T. asahii*. Morphological methods combined with biochemical methods were used most commonly (96/135, 71.1%). The antigen inhalation test was the identification method used for the five patients with SHP (Fig. 2d). Among the 135 cases, 128 patients (128/135 94.8%) were tested by using morphological methods (API 20C AUX yeast identification system, ID 32C yeast identification system, Vitek automatic microbial identification system). Biochemical techniques were used in 119 cases (119/135, 88.1%) and 24 cases (24/135, 18.4%) used a genetic method (LSU, D1/D2, ITS region nucleic acid sequence determination).

### *In vitro* susceptibility test

Of the 140 patients with *T. asahii* infection, 91 had an *in vitro* susceptibility test, consisting of 10 cases from 1996 to 2005 and 81 cases from 2006 to 2019. Among the 10 patients who had an *in vitro* susceptibility test from 1996 to 2005, four of them were sensitive to the antifungal drug voriconazole. Of the 81 patients who had *in vitro* susceptibility test between 2006 and 2015, 41 were sensitive to voriconazole, accounting for 37.3% of all patients (Table 4). Among *in vitro* susceptibility test methods, the M27-A2, M27-A3 and E-test methods were used widely on *T. asahii* infection patients.

**Table 4.** Drug effectiveness by *in vitro* drug sensitivity test in 140 patients^a^ with *T. asahii* infection

Drug	1996–2005	2006–2019
	No. (%）	No. (%）
Amphotericin B	1 (10)	24 (21.8)
Voriconazole	4 (40)	41 (37.3)
Itraconazole	3 (30)	19 (17.3)
Miconazole	0 (0)	1 (0.9)
Ketoconazole	1 (10)	0 (0)
Fluconazole	1 (10)	25 (22.7)
Total	10 (100.0)	110 (100.0)

a Some patients were sensitive to more than one drug.

### Drug selection

Regarding treatment and medication options for *T. asahii* infection, the ESCMID/ECMM guidelines recommended the use of voriconazole for the treatment of trichosporosis in 2014. The guidelines were based on the results of *in vitro* susceptibility testing, animal model testing and supporting evidence from a few case reports of Trichosporon infection [49]. However, more than 50 species of Trichosporon have been identified currently and different species of Trichosporon exhibit different levels of sensitivity to drugs. For example, the MIC of amphotericin B for *T. asahii*, *T. faecale* and *T. coremiiforme* was relatively higher, than that for *T. inkin*, *T. ovoides*, *T. japonicum*, *T. domesticum*, *T. montevideense* and *T. cutaneum* [46, 53–55]. Therefore, this guideline has a limited reference value for the usage of voriconazole in treatment of *T. asahii* infection.

**Fig. 3.**
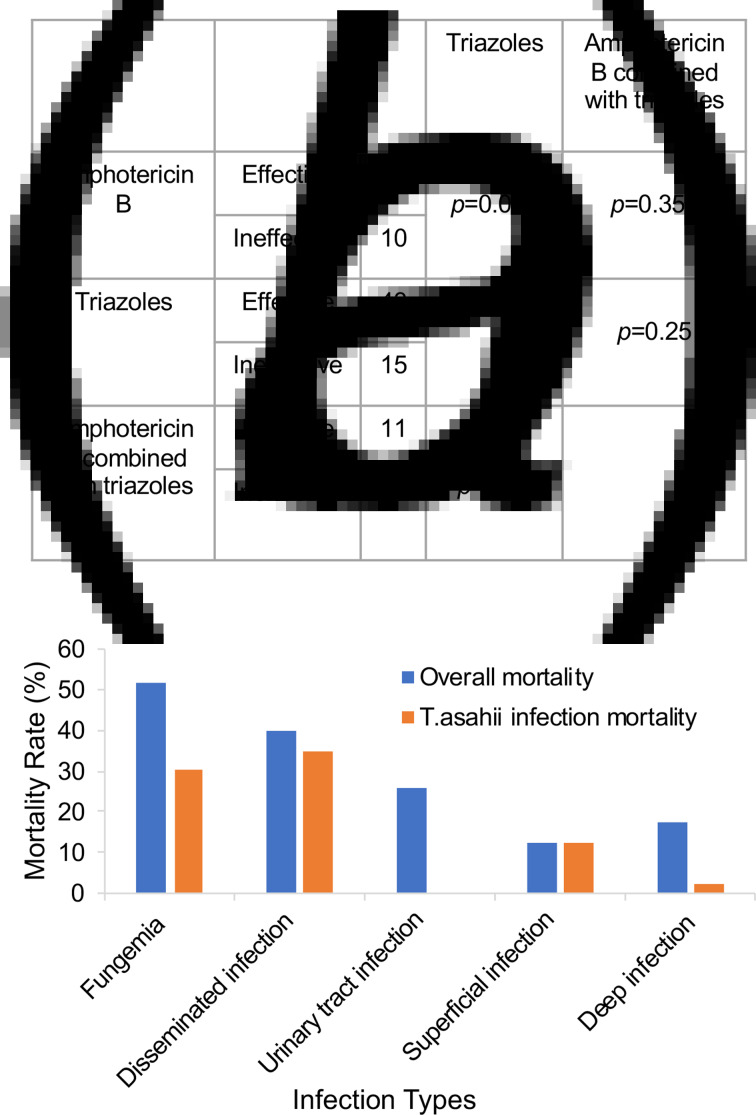
Statistical analysis of drug susceptibility testing and clinical outcome. (a) *In vitro* drug susceptibility testing of triazoles and amphotericin B. The *P*-value was calculated by using Fisher's exact test. (b) Mortality rate of different infection types.

Of the 140 patients with *T. asahii* infections, the treatment strategies were complex (Fig. 3a). The efficiency of single-drug therapy of amphotericin B was 70.6%, whereas the efficiency of triazole in antifungal therapy was 74.1%. The difference between the two groups was statistically significant (*P* = 0.015). However, the efficacy of a combined dosage of amphotericin B and triazole was reduced to 57.9%. There was no significant difference between the combination group and the single drug group (*P* > 0.05), but the efficacy of triazole in anti-*T. asahii* infection was better than amphotericin B (*P* < 0.05).

### Outcomes

Previous studies have shown that the mortality rate of Trichosporon infection can range from 53% to 80% [48, 61, 63, 64], but these studies only examined the mortality rate, and did not analyse the specific cause of death. They failed to answer questions such as whether the patient died from Trichosporon infection or other underlying diseases or if the main cause of death was due to complications resulting from the underlying disease. To clarify the mortality rate due to *T. asahii* infection, we excluded patients who died from underlying diseases and other causes of death. Only patients with death caused by *T. asahii* infection were considered. Of the 140 patients with *T. asahii* infection, the overall mortality rate of 140 patients was 30.0%, and the mortality rate caused by *T. asahii* infection was 13.6%. Furthermore, mortality was different in patients with varying infection types of *T. asahii*. Fungaemia was associated with the highest mortality rate, 51.5%, including 30.3% caused by *T. asahii* infection. This was followed by disseminated infection at 40.0%. Of note, 35% of deaths were caused solely by *T. asahii* infection (Fig. 3b).

### Comparative analysis of cases in China and other countries

**Fig. 4.**
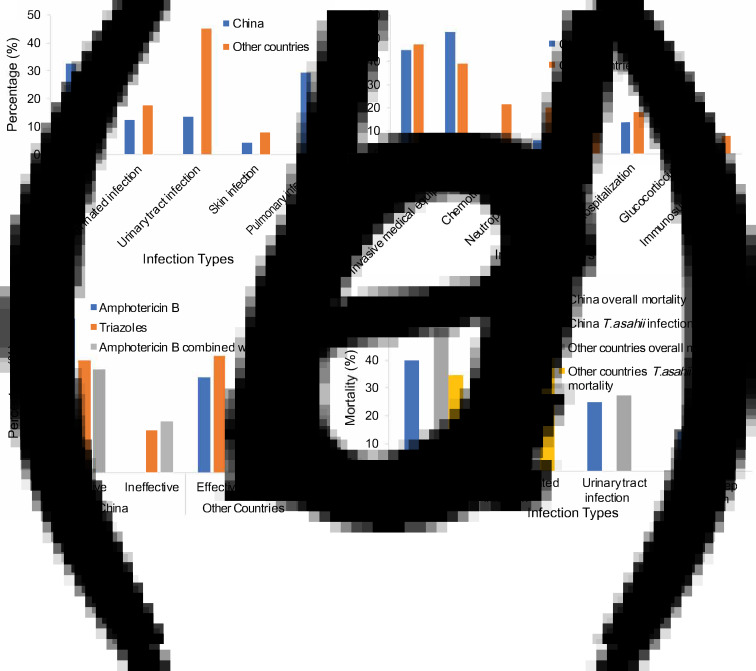
Comparative analysis of 51 Chinese patients and 89 patients in other countries. Comparative distributions were shown for (a) infection types, (b) inducing factors, (c) drug sensitivity and (d) mortality.

Since there were a large number of patients reported in China, we also statistically compared the characteristics of Chinese patients with those of other countries. We found that fungaemia and pulmonary infection in Chinese patients was higher than in other countries (>3-fold). On the other hand, the number of patients with urinary tract infections in other countries was greater compared to China (>3-fold) (Fig. 4a). For risk factors, we confirmed that antibiotic treatment and invasive medical device users were the most common in both cohorts. However, the number of patients with risk factors such as chemotherapy, neutrophil reduction and bacterial infection was greater in other countries compared to China (>3-fold) (Fig. 4b).

For the methods of identification and diagnosis, both China and other countries used standard morphological and biochemical methods (>50%). The results of the *in vitro* drug sensitivity test were also very similar. Comparisons of clinical drug usage showed no difference, and both groups preferred triazoles and amphotericin B (Fig. 4c). However, the overall mortality rate reported in Chinese patients was lower (21.6%) than that of other countries (34.8%) (Fig. 4d).

## Discussion

This study investigated 140 global *T. asahii* infection cases reported within the past 23 years. For statistical and comparative analysis, we used critical criteria to select both English and Chinese reports of *T. asahii* infections. We found that the number of *T. asahii* infections showed a dramatic increase from 2006 to 2015 (107 cases) in Asia. We also found that the patients in this study mainly used triazole antifungal drugs or amphotericin B alone, or the combination of triazoles and amphotericin B. The results showed that the antifungal effect of triazoles, such as voriconazole, fluconazole and itraconazole was the most effective in the treatment of *T. asahii* infection. This is consistent with the guidelines developed by ESCMID/ECMM in 2014. Furthermore, we found that the combined usage of amphotericin B and triazole was less effective than individual triazole drug usage. Therefore, our results suggest that the first recommended drug for the treatment of *T. asahii* infection should be a triazole. The statistical ranking of drug sensitivities based on *in vitro* susceptibility test (Table 4) suggests that voriconazole would be the first choice for treatment, followed by fluconazole, amphotericin B and itraconazole.

Since many of *T. asahii* infections were reported in Chinese, we further collected and investigated 51 Chinese cases. Through the comparison of cases between China and other countries, we found that although they are very similar for a majority of the criteria studied, there are still many differences. In particular, fungaemia and pulmonary infections were greater in Chinese patients, but urinary tract infections had a lower frequency in Chinese patients than in cases from other countries. Due to the strict inclusion criteria in this study, the actual incidence of *T. asahii* infections in the world is greater than 140 cases. Therefore, a more accurate epidemiological analysis of *T. asahii* infection requires collaboration with researchers from all over the world. We found that many types of clinical information, such as the infection process and the source of their pathogens, are missing. But this information could be valuable for understanding the infection process and mechanism of *T. asahii*. For example, a recent time series study of breakthrough IFIs [62] defined different clinic trail patterns of breakthrough IFI. A similar analysis can be performed for *T. asahii* infections if detailed and time series clinic records can be amassed. Meanwhile, acting as a reference for the clinical drug selection to treat *T. asahii* infection, our study can be improved by precisely controlling confounding factors including weight, vital organ function, etc. that were inaccessible now. Therefore, specific information such as the appropriate therapeutic dose, maintenance dose and duration of treatment remain to be determined.

## Data Availability

The authors confirm that the data supporting the findings of this study are available within the article.
